# Respiratory viruses in children hospitalized for acute lower respiratory tract infection in Ghana

**DOI:** 10.1186/1743-422X-9-78

**Published:** 2012-04-10

**Authors:** Theophilus B Kwofie, Yaw A Anane, Bernard Nkrumah, Augustina Annan, Samuel B Nguah, Michael Owusu

**Affiliations:** 1 School Of Medical, Sciences, Kumasi, Department of Clinical Microbiology Kwame, Nkrumah University of Science and Technology (KNUST), Kumasi, Ghana; 2 Kumasi Centre for Collaborative Research in Tropical Medicine, Kumasi, Ghana; 3 Department of Child Health, Komfo Anokye Teaching Hospital, Kumasi, Ghana

**Keywords:** Respiratory Viruses, Hospitalized children, Real-Time PCR

## Abstract

**Background:**

Acute respiratory tract infections are one of the major causes of morbidity and mortality among young children in developing countries. Information on the viral aetiology of acute respiratory infections in developing countries is very limited. The study was done to identify viruses associated with acute lower respiratory tract infection among children less than 5 years.

**Method:**

Nasopharyngeal samples and blood cultures were collected from children less than 5 years who have been hospitalized for acute lower respiratory tract infection. Viruses and bacteria were identified using Reverse Transcriptase Real-Time Polymerase Chain Reaction and conventional biochemical techniques.

**Results:**

Out of 128 patients recruited, 33(25.88%%, 95%CI: 18.5% to 34.2%) were positive for one or more viruses. Respiratory Syncytial Virus (RSV) was detected in 18(14.1%, 95%CI: 8.5% to 21.3%) patients followed by Adenoviruses (AdV) in 13(10.2%, 95%CI: 5.5% to 16.7%), Parainfluenza (PIV type: 1, 2, 3) in 4(3.1%, 95%CI: 0.9% to 7.8%) and influenza B viruses in 1(0.8%, 95%CI: 0.0 to 4.3). Concomitant viral and bacterial co-infection occurred in two patients. There were no detectable significant differences in the clinical signs, symptoms and severity for the various pathogens isolated. A total of 61.1% (22/36) of positive viruses were detected during the rainy season and Respiratory Syncytial Virus was the most predominant.

**Conclusion:**

The study has demonstrated an important burden of respiratory viruses as major causes of childhood acute respiratory infection in a tertiary health institution in Ghana. The data addresses a need for more studies on viral associated respiratory tract infection.

## Background

Acute respiratory infections (ARI) are one of the major causes of morbidity and mortality in young children throughout the world especially in developing countries [1,2]. Data from WHO estimated the burden of ARI at 94,037,000 disability-adjusted life years (DALYs) and 3.9 million deaths in 2001 [3]. Similar report from a meta-analysis study demonstrates that throughout the world 1.9 million (95% CI 1.6-2.2 million) children died from ARI in 2000, 70% of them in Africa and Southeast Asia [2]. A further systematic analysis also estimated 1.575 million (uncertainty range: 1.046 million - 1.874 million) deaths of children worldwide in 2008 as due to ARI [4].

Majority of acute lower respiratory tract infections(ALRTI) in developed countries have been reported to be often due to viral pathogens of which most common are RSV, PIV, influenza viruses, Adv, human Coronaviruses and Bocaviruses [5-7]. On the contrary, information on these viruses in developing countries is limited probably due to paucity of modern diagnostic molecular techniques. These infections are therefore treated unsuccessfully with antibiotics based on suspicion of bacterial causes [8].

Apart from the public health concern of nosocomial infections that are associated with viral respiratory infections [9,10], significant costs derived from long duration of hospitalization and several healthcare visits could also aggravate the poor socio-economic status and increased child mortality in developing countries including Ghana.

Lessons from the outbreak of the Severe Acute Respiratory Syndrome (SARS) epidemics which resulted in the death of 776 individuals [11] and the recent emergence of a novel swine flu pandemic emphasize the risk posed by respiratory viral infections in humans [12].

This study was done to determine the burden of respiratory viruses among children hospitalized at the Komfo Anokye Teaching Hospital for acute lower respiratory illness using the Real Time Polymerase Chain Reaction (RT-PCR).

## Methodology

### Study design

This was a hospital based cross-sectional study of children less or equal to five years and hospitalized for acute lower respiratory tract illness.

### Study site

The study was performed at the children's ward of the Komfo Anokye Teaching Hospital (KATH), Ghana from January to December 2008. KATH is approximately a thousand bed tertiary medical facility located in Ashanti Region, Kumasi, the confluence of the transportation network in the central part of Ghana. Its position makes it the most accessible tertiary and referral medical facility in Ghana attending to a population of over 4.4 million in the Ashanti region and beyond [13].

### Sampling

To have a year round incidence of the various viral agents, recruitment was done throughout the year 2008. Screening and recruitment were started at the beginning of every week till two or three patients were recruited. After a maximum of three patients have been obtained it was suspended till the beginning of the next week. This cycle was repeated till the maximum of 11 patients were recruited for each month.

At the recruitment station all patients arriving at the unit were screened but only those less than five completed years were recruited. Patients recruited into the study should have features of severe pneumonia or very severe pneumonia as defined by the WHO-IMCI (World Health Organisation Integrated Management of Childhood Illness) protocol [14,15]. Severe pneumonia was said to be present if the child has a history of cough and/or difficult breathing of less than 3 weeks duration, with lower chest wall recession. Severe pneumonia in addition to cyanosis and/or inability to feed or drink was classified as very severe pneumonia. A child was recruited into the study only after the guardian or parents had consented after the objectives of the study had been explained in English or the local dialect. A standardized case record form was then used to record the history of the illness as well as the presenting clinical features of pneumonia.

Five (5 ml) of blood sample was then taken into 25 ml of Brain Heart Infusion Broth (BHIB) and quickly sent to the bacteriological laboratory for culture. Nasopharyngeal specimens were also taken using the nasopharyngeal flocked swab (Copan, Italy). The swab was gently inserted up the nostril towards the pharynx until resistance was felt and then rotated 3 times to obtain epithelial cells. It was then withdrawn and put into 2.5 ml phosphate buffered saline (X1). The samples were transported on ice to the laboratory within few hours of collection. They were then vortexed, transferred into 1.5 ml eppendorf tubes (Eppendorf, Germany) and kept at -80°C for Polymerase Chain Reaction (PCR). Both samples were taken by experienced prior trained nurses of the children ward. Children were excluded if both samples could not be obtained for any reason.

### Viral identification

Viral nucleic acids were extracted from samples using QIAamp viral mini kit (Qiagen, Germany) according to the manufacturers' instruction [16]. RT-PCR amplification was performed for the detection of Adv, RSV, Influenza A (Flu A), Influenza B (Flu B), Parainfluenza 1(PIV 1), Parainfluenza 2 (PIV 2) and Parainfluenza 3 (PIV 3) using the light cycler version 1.5 (Roche, Germany). Primers highly specific to each of the viruses were used (Table 1). The reaction mixture for RNA viruses was made up of 5 μl of RNA extract, 1 μl deoxynucleotide (dNTP) triphosphate (10 mM) (Qiagen), 1 μl Bovine Serum Albumin (BSA)(1 mg/ml)(Qiagen), 12.5 μl Qiagen One Step RT-PCR Buffer x5 (containing 12.5 mM MgCl_2_) (Qiagen), 1 μl Qiagen One Step Enzyme Mix, 1 μl each of sense and antisense primer(10 μM each), 0.5 μl Probe and 2.0 μl of RNase free water. DNA virus reaction mixture was made up of 5 μl PCR buffer (10X), 1 μl of dNTP mix (10 mM each), 1 μl of MgCl_2 _(50 mM), 0.5 μl of Hot Star Taq DNA Polymerase, 0.5 μl of probe, 1 μl of BSA (1 mg/ml) and 1 μl each of sense and antisense primers.

**Table 1:** Target genes and primer sequences for respiratory pathogens

Virus	Target gene	Function	Oligonucleotide sequence
RSV^a ^(A/B)	Matrix gene [17]	Forward primer	5'-GGAAACATACGTGAACAAGCTTCA

		Reverse primer A	5'-CATCGTCTTTTTCTAAGACATTGTATTGA

		Reverse primer B	5'-TCATCATCTTTTTCTAGAACATTGTACTGA

		Probe	6FAM-TGTGTATGTGGAGCCTT- MGBNFQ

Adenovirus	Hexon gene [18]	Forward primer	5'-GCCACGGTGGGGTTTCTAAACTT

		Reverse primer	5'-GCCCCAGTGGTCTTACATGCACAT

		Probe	6FAM-TGCACCAGACCCGGGCTCAGGTACTCCGA-TAMRA

PIV I^b^	Polymerase gene [19]	Forward primer	5'-ACAGATGAAATTTTCAAGTGCTACTTTAGT

		Reverse primer	5'-GCCTCTTTTAATGCCATATTATCATTAGA

		Probe	6FAM-ATGGTAATAAATCGACTCGCT- MGBNFQ

PIV II^c^	Polymerase gene [19]	Forward primer	5'-TGCATGTTTTATAACTACTGATCTTGCTAA

		Reverse primer	5'-GTTCGAGCAAAATGGATTATGGT

		Probe	6FAM-ACTGTCTTCAATGGAGATAT- MGBNFQ

PIV III^d^	Matrix gene [19]	Forward primer	5'-TGCTGTTCGATGCCAACAA

		Reverse primer	5'-ATTTTATGCTCCTATCTAGTGGAAGACA

		Probe	6FAM-TTGCTCTTGCTCCTCA- MGBNFQ

Influenza A	Matrix gene [20]	Forward primer A1	5'-GGACTGCAGCGTAGACGCTT

		Forward primer A2	5'-CATCCTGTTGTATATGAGGCCCAT

		Reverse primer	5'-CATTCTGTTGTATATGAGGCCCAT

		Probe	6FAM-CTCAGTTATTCTGCTGGTGCACTTGCCA- MGBNFQ

Influenza B	Hemagglutinin (HA)gene [20]	Forward primer	5'-AAATACGGTGGATTAAATAAAAGCAA

		Reverse primer	5'-CCAGCAATAGCTCCGAAGAAA

		Probe	6FAM-CACCCATATTGGGCAATTTCCTATGGC- MGBNFQ

RSV^a^, Respiratory syncytial virus; PIV I^b^, Parainfluenza virus type 1; PIV II^c^, Parainfluenza virus type 2, PIV III^d^, Parainfluenza virus type 3.

The PCR conditions for PIV, RSV and Influenza viruses consisted of an initial reverse transcription at 20 minutes for 50°C, Taq DNA polymerase activation at 95°C followed by forty five (45) cycles of denaturation at 15 seconds for 95°C and annealing at 15 seconds for 58°C. The initial reverse transcription step was omitted in the case of Adv.

### Controls and standards

For each batch of tests that were run, RNase free water (Qiagen, Germany) was used as negative control and i*n vitro *transcribed gene of the various viruses was used to determine the detection limit of the assay as positive control. The *in vitro *transcripts were prepared in our collaborative institution (Bonn Institute of Virology, Bonn, Germany) and transported to Ghana via cooling chain. The *in vitro *transcripts were prepared by amplifying the genes of interest of the viruses with specific set of primers. The products were ligated to the PCR2.1 vector using the TOPO TA cloning kit (Invitogen Corp, Carlsbad, CA) and transformed into competent E.coli cells. After overnight incubation, desirable colonies were selected and purified using Qiamp Spin-Miniprep Kit (Qiagen, Germany). PCR was performed on the purified product and RNA transcripts were then synthesized from the lowest dilution (with clear band) using Ambion Megascripts T3 kit (Invitogen corp, Calsbad, CA). The final products were purified using Rneasy Mini Kit (Qiagen, Germany) and the concentrations of the transcripts were determined using a spectrophotometer (Themoscientific, U.S.A). The copy numbers of the RNA transcripts were determined following the method of Fronhoffs [21]. Ten-fold dilutions of the RNA transcripts were prepared in Carrier RNA-RNase free water (310 μg of Carrier RNA in 310 ml of RNase free water) and tested. The detection limit was determined as the last dilution after which all other replicates gave negative results. The detection limits were Respiratory Syncytial Virus (RSV): 20 copies/μl, PIV 1: 1 - 78 copies/μl, PIV 2: 400 copies/μl, PIV 3: 30 copies/μl, Influenza A: 120 copies/μl and Influenza B: 480 copies/μl.

To determine the specificities, our assays were tested on other different viruses and they turn out negative.

### Bacteria identification

Bacterial isolates were identified using conventional biochemical methods including urease and indole production, citrate utilization, hydrogen sulphide, gas production and fermentation of sugars. The biochemical media used included Simon's Citrate medium, Urea and Triple Sugar Iron agar (TSI). Coagulase tests were performed for all staphylococci organisms.

### Statistical analysis

Data obtained was double entered into a spreadsheet database prepared with Microsoft^® ^Excel. It was then compared and cleaned for abnormal wrongful entries. Statistical analysis was done using STATA SE statistical software version 11.2(Texas, USA) after the data had been imported. Categorical variables such as age groups and their association with respiratory agents were analyzed using the Fischer's exact test. Continuous variables were expressed as medians with their inter-quartile ranges. A non-parametric K-sample test on the equality of medians was used to evaluate the differences in the medians of the various subgroups of the continuous variables. For all analysis done, a p-value of less than 0.05 was considered statistically significant.

### Ethical approval

The study protocol was approved by the Committee for Human Research, Publications and Ethics (CHRPE) of KATH and School of Medical Sciences, KNUST, Kumasi, Ashanti region, Ghana.

## Results

A total of 128 patients were recruited over the period of January to December 2008. The median age of the patients was 12 months (IQR: 6-24 months) and the number of males was 81(63.3%). Sixty one (47.7%) had severe pneumonia with the rest diagnosed as very severe pneumonia. At least one respiratory virus was detected in 33 (25.7%, 95%CI: 18.5% to 34.2%) children. Multiple viral infections were detected in 2 patients. One had an RSV and PIV 1 co-infection whiles the other had a combination of RSV, Influenza B and Adenovirus co-infection. Bacteria was isolated from only 12 (9.4%, 95%CI: 4.9 to 15.8%) patients with 10 of these being *Staphylococcus aureus*. The other two were *Klebsiella *species and *Coliform*. RSV and *Staphylococcus aureus *coinfection was also identified in two patients.

Six (20.0%) out of 30 children less than six months of age were found to be positive for viral infection (Table 2). The highest number of children infected were between 6 months and 2 years, the proportions infected for the various age groups were however not significantly different.

**Table 2:** Association between age groups and respiratory virus infection

	Age (months)			
	**≤ 5**	**6 to 23**	**24 to 60**	***p*-value**

	**(n = 30)**	**(n = 59)**	**(n = 39)**	

**Infections**				

Patients infected with at least one virus, no.(%)	6(20.0)	18(30.5)	9(23.1)	0.549

Respiratory syncytial virus, no (%)	4(13.3)	9(15.3)	5(12.8)	1.000

Adenovirus, no. (%)	2(6.7)	8(13.6)	3(7.7)	0.596

Parainfluenza 1, no. (%)	0 (0.0)	1(1.7)	1 (2.6)	1.000

Parainfluenza 3, no. (%)	0 (0.0)	2(3.4)	1(2.6)	0.798

Influenza B, no.(%)	0 (0.0)	1(1.7)	0 (0.0)	1.000

Clinical presentation of patients were compared to general pathogens identified (Table 3) and the individual viral pathogens (Table 4). Generally, there were no detectable significant differences in the clinical presentation as well as the gender and duration of illness of patients for the various pathogens isolated.

**Table 3:** Distribution of clinical presentation and microbial isolation

	Microbial Isolation					
**Presentation**	**None** **(n = 85)**	**Virus** **(n = 31)**	**Bacteria** **(n = 10)**	**Bacteria & Viruses** **(n = 2)**	**Total** **(n = 128)**	*p-value

Age(months) median(IQR)	12(5-25)	11(6-19)	17(8-24)	48(36-60)	128(100%)	0.282

Temperature median(IQR)	38(37.8-38.5)	38(37.7-38.6)	37.8(37.4-38.0)	37.8(37.4-38)	128(100%)	0.134

Duration of illness (days) median(IQR)	4 (3-6)	4(3-5)	6.5(4-14)	4.5(4-5)	4 (3-6)	0.187

Sex (male) n(%)	54(63.3)	20(64.5)	5(50.0)	2(100.0)	81(63.3)	0.668

Tachypnoea n (%)	61(71.8)	18(58.1)	6(60.0)	1(50.0)	86(67.2)	0.382

Chest recessions n (%)	80(94.1)	29(93.6)	9(90.0)	2(100.0)	120(93.4)	0.751

Wheeze n (%)	31(36.5)	14(45.2)	3(30.0)	2(100.0)	50(39.1)	0.268

Cyanosis n (%)	3(3.4)	3(5.5)	0 (0.0)	1(50.0)	7(5.3)	0.07

Poor feeding n (%)	48(56.5)	10(32.3)	3(30.0)	0	61(47.7)	**0.029**

Diarrhea n (%)	14(16.5)	4(12.9)	3(30.0)	0	21(16.4)	0.606

Vomiting n (%)	25(29.4)	8(25.8)	1(10.0)	1(50.0)	35(27.3)	0.461

Severe Pneumonia n (%)	35(41.2)	18(58.1)	7(70.0)	1(50.0)	61(47.7)	0.142

Very severe Pneumonia n (%)	50(58.8)	13(41.9)	3(30.0)	1(50.0)	67(52.3)	0.142

* *p*-values determined assuming the null hypothesis that the proportions of the clinical presentations are uniform among the various subgroups (i.e. none, viral, bacterial or bacterial and viral infections) for categorical variables or their medians for continuous clinical variables

**Table 4:** Clinical presentations of patients infected with individual respiratory viruses

		Respiratory Viruses			
**Clinical Presentation**	**RSV** **(n = 18)**	**Adv** **(n = 13)**	**PIV-1** **(n = 1)**	**PIV-3** **(n = 3)**	**Flu B** **(n = 1)**

Temperature median(IQR)	38(37.7-38.2)	37.9(37.4-38.5)	37.5(37.5-37.5)	38.6(38.2-39)	38.8(38.8-38.8)

Duration of illness median(IQR)	4(3-5)	3 ((2-6)	4(4-4)	3((2-4)	6(6-6)

Sex (male) n(%)	13(72.2)	9(69.2)	1(100.0)	1(33.3)	1(100)

Tachypnoea n(%)	11(61.1)	7(53.9)	0	2(66.7)	1(100)

Chest recessions n(%)	17(94.4)	12(92.3)	1(100.0)	3(100)	1(100)

Wheeze n(%)	9(50.0)	8(61.5)	0	3(100)	1(100)

Cyanosis n(%)	1(5.6)	3 (23.1)^a^	0	3(100)	1(100)

Poor feeding n(%)	8(44.4)	1(7.7)^b^	0	1(33.3)	0

Diarrhea n(%)	1(5.6)	1(7.7)	0	3(100)	0

Vomiting n(%)	6 (33.3)	1(7.7)	0	3(100)	0

Severe Pneumonia n(%)	9(50.0)	9(69.2)	1(100.0)	3(100)	1(100)

Very severe Pneumonia n (%)	9(50.0)	4(30.8)	0	3(100)	0

RSV; Respiratory syncytial virus; PIV-I, Parainfluenza virus type 1, PIV-III; Parainfluenza virus type 3,Adv; Adenovirus, Flu B; Influenza B. Parainfluenza type 2 and Influenza A were not detected in clinical samples. ^a^P = 0.03 vs patients having cyanosis and infected with adenovirus. ^b^P = 0.02 vs patients who were poorly fed with adenovirus infection.

**Figure 1:**
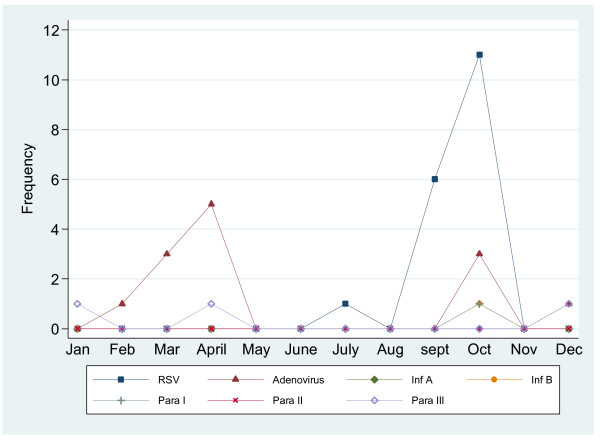
**Monthly distribution of viruses**. The figure describes the various viruses identified from January to December 2008. RSV: Respiratory Syncytial Viruses; Inf A: Influenza A virus; Inf B: Influenza B virus; PARA 1: Parainfluenza type 1 virus; PARA II: Parainfluenza type 2 virus; PARA III: Parainfluenza type 3 virus.

The study also investigated the seasonal variation of viruses. RSV infections were detected throughout the year however the peak infection rate occurred during the minor rainy seasons (October) (Figure 1). Adenovirus infections on the other hand had two main peaks occurring in the month of April and October.

## Discussion

Viral agents play an important role in acute lower respiratory infections and may herald the onset of pneumonia caused by secondary bacterial infections [22]. Although information on the causes of respiratory illness in tropical countries is very scanty, available data indicates about one -third of the cases of respiratory tract infections are due to viruses [23].

The present study identified viruses in 25.8% of patients hospitalized for ALRTI with RSV being the most predominant. The overall prevalence is comparable to previous studies done in other developing countries [24] and the predominance of RSV is in accordance with the assertion that this virus is the single most frequent lower respiratory tract pathogen in infants and young children worldwide [25-27].

Adenoviruses, the second highest viruses detected in this study have been reported to be responsible for 5-10% of lower respiratory tract infections with the highest rate occurring in younger children [28-31]. Our study similarly recorded a 10.2% detection rate of Adenoviruses however there was no statistical difference in the age specific prevalence. This could possibly be due to the few number of younger children (less than one year) enrolled in this study.

This study generally recorded higher cases of tachypnoea, chest recessions and very severe pneumonia in RSV compared to Adenovirus infected patients but the differences were not statistically significant. Although similar clinical presentations have been found to be associated with RSV [32,33], our small sample size could account for our inability to detect these differences.

Among patients who were poorly fed, those without viral or bacterial infections were found to be higher (p = 0.03). Poor feeding and malnutrition has generally been associated with less risk for respiratory viral infection in developing countries [34-36] however the possibility of increased mortality could occur especially when bacterial infections are involved. Further case controlled studies are therefore needed in developing countries to investigate this observation

There have been fewer studies of PIV infections in developing countries and most of them do not differentiate the subtypes of the viruses. Our study recorded a 3.1% prevalence of PIV infections with the predominant type being PIV-3. Similar hospital based studies have been reported in other developing countries [37-40]. PIV infections have been strongly associated with croup [41,42]. The present study recorded no case of croup among PIV patients.

Viral associated bacteremia was detected in two patients (1.7%). Low rate of secondary bacterial infections has generally been reported in developing countries with the isolation rate varying between 2 and 10% [43-45]. Studies in some developed countries however reported the converse [46,47] probably due to the higher sensitivity of the bacteria identification techniques used. The blood culture identification system used in this study may be limited in the detection of bacteria associated with lower respiratory tract infection. Perhaps the detection rate could have increased if the present study had identified bacterial pathogens from nasopharyngeal aspirates or washings as reported by Hishinki et al. [47].

The isolation of *Staphylococcus aureus *in an RSV positive patient in the present study was similar to studies reported by Berman et al., [48] and Cherian et al., [43]. In their case, the bacterium was associated with fatality in the children.

The present study observed that all subjects enrolled in this study were treated empirically with antibiotics in accordance with the paediatric emergency treatment protocol for managing severe and very severe pneumonia. Similar occurrences have been reported where patients were treated unsuccessfully with multiple antibiotics [8]. Policy makers may therefore consider reviewing the clinical algorithm for patient management as the economic cost both to the health service and the patient's household derived from the use of antibiotics could be enormous.

The period of this study experienced major rainfalls in the months of May to July and minor rainfalls in the month of September to October. The month of October recorded the highest detection of viral infections (57.6%; 19/33) with the commonest being RSV. This phenomenon has been reported in other developing countries [43,49]. The occurrence of RSV infections in the rainy and cold season could be due to overcrowding as a result of populations staying more to their homes. More studies are however needed to define the seasonality of respiratory viruses in tropical countries.

Our study had some limitations which include underestimation of the overall prevalence of respiratory viruses since we did not test for coronaviruses, human metapneumovirus, bocaviruses and rhinoviruses which have also been reported in hospitalized patients. Also the negative results recorded for Influenza A and PIV 2 and the low prevalence of influenza B in our samples could be due to the low sensitivity of the assay for these viruses. The limit of detections of Influenza A, Influenza B and PIV 2 are 120 copies/μl, 480 copies/μl and 400 copies/μl respectively as such viral copies below these limits of detections could be missed by our assay.

## Conclusion

This study has demonstrated that respiratory viruses are associated with a considerable number of hospital admissions in Ghana. Hospitalized children presenting with symptoms of ALRTI may not necessarily need treatment with antibiotics but rather antiviral drug options could be explored. The importance of further cross-sectional and longitudinal studies of viral-associated respiratory infections among out-patients and healthy populations cannot be over emphasized. This will contribute immensely to overcoming the paucity of data on the importance of respiratory associated viruses to the burden of disease and the morbidity and mortality of under five children.

## Abbreviations

Adv: Adenoviruses; ALRTI: Acute lower Respiratory Tract Infection; ARI: Acute Respiratory Tract Infection; BHIB: Brain Heart Infusion Broth; BSA: Bovine Serum Albumin; CHRPE: Committee for Human Research Publications and Ethic; DALYs: Disability Adjusted Life Years; Flu A: Influenza A; Flu B: Influenza B; KATH: Komfo Anokye Teaching Hospital; KNUST: Kwame Nkrumah University Of Science and Technology; PCR: Polymerase Chain Reaction; PIV: Parainfluenza Virus; RSV: Respiratory Syncytial Virus; SARS: Severe Acute Respiratory Syndrome; TSI: Triple Sugar Iron agar; WHO-IMCI: (World Health Organisation Integrated Management of Childhood Illness).

## Competing interests

The authors declare that they have no competing interests.

## Authors' contributions

YAA, AA and BN co-worked on the data collection and contributed to laboratory analysis of the samples. SBN performed statistical analysis of the data and contributed to writing of the manuscript. TBK planned, initiated the study and contributed to writing of the manuscript. OW carried out the laboratory analysis of the samples and contributed to writing of the manuscript and interpretation of the data. All authors have read and approved the manuscript.

## References

[B1] Denny FW Loda FA Acute respiratory infections are the leading cause of death in children in developing countries Am J Trop Med Hyg 1986 35 1 1 2 3946732 10.4269/ajtmh.1986.35.1

[B2] Williams BG Gouws E Boschi-Pinto C Bryce J Dye C Estimates of world-wide distribution of child deaths from acute respiratory infections Lancet Infect Dis 2002 2 1 25 32 10.1016/S1473-3099(01)00170-0 11892493

[B3] World Health Organisation Burden of disease in DALYs by sex and mortality stratum in WHO regions, estimates for 2001 The world health report 2002 192 197

[B4] Black RE Cousens S Johnson HL Lawn JE Rudan I Bassani DG Jha P Campbell H Walker CF Cibulskis R Global, regional, and national causes of child mortality in 2008: a systematic analysis Lancet 2010 375 9730 1969 1987 10.1016/S0140-6736(10)60549-1 20466419

[B5] Meerhoff TJ Mosnier A Schellevis F Paget WJ EISS RSV Task Group Progress in the surveillance of respiratory syncytial virus (RSV) in Europe: 2001-2008 Euro Surveill 2009 14 40 pii = 19346 19822120

[B6] Bellau-Pujol S Vabret A Legrand L Dina J Gouarin S Petitjean-Lecherbonnier J Pozzetto B Ginevra C Freymuth F Development of three multiplex RT-PCR assays for the detection of 12 respiratory RNA viruses J Virol Methods 2005 126 1-2 53 63 10.1016/j.jviromet.2005.01.020 15847919 PMC7112904

[B7] Iwane MK Edwards KM Szilagyi PG Walker FJ Griffin MR Weinberg GA Coulen C Poehling KA Shone LP Balter S Population-Based Surveillance for Hospitalizations Associated With Respiratory Syncytial Virus, Influenza Virus, and Parainfluenza Viruses Among Young Children Pediatrics 2004 113 6 1758 1764 10.1542/peds.113.6.1758 15173503

[B8] Breese Hall C Powell KR Schnabel KC Gala CL Pincus PH Risk of secondary bacterial infection in infants hospitalized with respiratory syncytial viral infection The Journal of pediatrics 1988 113 2 266 271 10.1016/S0022-3476(88)80263-4 3397789

[B9] Weber MW Dackour R Usen S Schneider G Adegbola RA Cane P Jaffar S Milligan P Greenwood BM Whittle H The clinical spectrum of respiratory syncytial virus disease in The Gambia Pediatr Infect Dis J 1998 17 3 224 230 10.1097/00006454-199803000-00010 9535250

[B10] Bisno A BARRATT N Swanston W Spence L An outbreak of acute respiratory disease in Trinidad associated with para-influenza viruses Am J Epidemiol 1970 91 1 68 4313416 10.1093/oxfordjournals.aje.a121114

[B11] Kuiken T Fouchier RAM Schutten M Rimmelzwaan GF van Amerongen G van Riel D Laman JD de Jong T van Doornum G Lim W Newly discovered coronavirus as the primary cause of severe acute respiratory syndrome Lancet 2003 362 9380 263 270 10.1016/S0140-6736(03)13967-0 12892955 PMC7112434

[B12] Novel SOIA Dawood F Jain S Finelli L Shaw M Lindstrom S Garten R Gubareva L Xu X Bridges C Emergence of a novel swine-origin influenza A (H1N1) virus in humans N Eng J Med 2009 360 25 2605 10.1056/NEJMoa0903810 19423869

[B13] Komfo Anokye Teaching Hospital, Centre of Excellence http://www.kathhsp.org/aboutus1.php

[B14] Tulloch J Integrated approach to child health in developing countries Lancet 1999 354 2 16 20 10.1016/s0140-6736(99)90252-0 10507254

[B15] Gove S Integrated management of childhood illness by outpatient health workers: technical basis and overview. The WHO Working Group on Guidelines for Integrated Management of the Sick Child Bull World Health Organ 1997 75 1 7 24 9529714 PMC2486995

[B16] Qiagen Protocol: Purification of Viral RNA (spin Protocol) Qiagen Viral RNA Mini Handbook 2005 2 Hilden, Germany 23 25

[B17] Fronhoffs S Totzke G Stier S Wernert N Rothe M Brüning T Koch B Sachinidis A Vetter H Ko Y A method for the rapid construction of cRNA standard curves in quantitative real-time reverse transcription polymerase chain reaction Mol Cell Probes 2002 16 2 99 110 10.1006/mcpr.2002.0405 12030760

[B18] Berman S Epidemiology of Acute Respiratory Infections in Children of Developing Countries Review of infectious diseases 1991 13 6 454 462 10.1093/clinids/13.Supplement_6.S454 1862276

[B19] Karaivanova GM Viral respiratory infections and their role as public health problem in tropical countries (review) Afr J Med Med Sci 1995 24 1 1 7 7495193

[B20] Weber MW Mulholland EK Greenwood BM Respiratory syncytial virus infection in tropical and developing countries Trop Med Int Health 1998 3 4 268 280 10.1046/j.1365-3156.1998.00213.x 9623927

[B21] Centers For Disease Control and Prevention Update: respiratory syncytial virus activity-United States, 1996-97 season MMWR Morb Mortal Wkly Rep 1996 45 48 1053 1055 8975116

[B22] Sung RY Chan RC Tam JS Cheng AF Murray HG Epidemiology and aetiology of acute bronchiolitis in Hong Kong infants Epidemiol Infect 1992 108 1 147 154 10.1017/S0950268800049591 1312477 PMC2272179

[B23] Sung CC Chi H Chiu NC Huang DT Weng LC Wang NY Huang FY Viral etiology of acute lower respiratory tract infections in hospitalized young children in Northern Taiwan J Microbiol Immunol Infect 2011 44 3 184 190 10.1016/j.jmii.2011.01.025 21524612 PMC7105033

[B24] Chen HL Chiou SS Hsiao HP Ke GM Lin YC Lin KH Jong YJ Respiratory adenoviral infections in children: a study of hospitalized cases in southern Taiwan in 2001-2002 J Trop Pediatr 2004 50 5 279 284 10.1093/tropej/50.5.279 15510759

[B25] Arden KE McErlean P Nissen MD Sloots TP Mackay IM Frequent detection of human rhinoviruses, paramyxoviruses, coronaviruses, and bocavirus during acute respiratory tract infections J Med Virol 2006 78 9 1232 1240 10.1002/jmv.20689 16847968 PMC7167201

[B26] Wong S Pabbaraju K Pang XL Lee BE Fox JD Detection of a broad range of human adenoviruses in respiratory tract samples using a sensitive multiplex real time PCR assay J Med Virol 2008 80 5 856 865 10.1002/jmv.21136 18360899 PMC7166731

[B27] Rodríguez C José A Daszenies C García M Meyer R Gonzales R Adenovirus pneumonia in infants and factors for developing bronchiolitis obliterans: a 5-year follow-up Pediatr Pulmonol 2006 41 10 947 953 10.1002/ppul.20472 16871594

[B28] Loscertales MP Roca A Ventura PJ Abacassamo F Santos FD Sitaube M Menendez C Greenwood BM Saiz JC Alonso PL Epidemiology and clinical presentation of respiratory syncytial virus infection in a rural area of southern Mozambique Pediatr Infect Dis J 2002 21 2 148 10.1097/00006454-200202000-00013 11840083

[B29] Mathisen M Strand TA Sharma BN Chandyo RK Valentiner-Branth P Basnet S Adhikari RK Hvidsten D Shrestha PS Sommerfelt H Clinical presentation and severity of viral community-acquired pneumonia in young Nepalese children Pediatr Infect Dis J 2010 29 1 e1 10.1097/INF.0b013e3181c2a1b9 19935451

[B30] Adegbola RA Falade AG Sam BE Aidoo M Baldeh I Hazlett D Whittle H Greenwood BM Mulholland EK The etiology of pneumonia in malnourished and well-nourished Gambian children Pediatr Infect Dis J 1994 13 11 975 982 10.1097/00006454-199411000-00008 7845751

[B31] Nwankwo MU Okuonghae HO Currier G Schuit KE Respiratory syncytial virus infections in malnourished children Ann Trop Paediatr 1994 14 2 125 130 7521627 10.1080/02724936.1994.11747704

[B32] Phillips P Lehmann D Spooner V Barker J Tulloch S Sungu M Canil K Pratt R Lupiwa T Alpers M Viruses associated with acute lower respiratory tract infections in children from the eastern highlands of Papua New Guinea (1983-1985) Southeast Asian J Trop Med Public Health 1990 21 3 373 1963705

[B33] Wafula EM Onyango FE Mirza WM Macharia WM Wamola I Ndinya-Achola JO Agwanda R Waigwa RN Musia J Epidemiology of Acute Respiratory Tract Infections Among Young Children in Kenya Review of infectious diseases 1990 12 8 1035 1038 10.1093/clinids/12.Supplement_8.S1035 2270401

[B34] Hazlett D Bell T Tukei P Ademba G Ochieng W Magana J Gathara G Wafula E Pamba A Ndinya-Achola J Viral etiology and epidemiology of acute respiratory infections in children in Nairobi, Kenya Am J Trop Med Hyg 1988 39 6 632 2849887 10.4269/ajtmh.1988.39.632

[B35] Forgie IM O'Neil KP Lloyd-Evans N Leinonen M Campbell H Whittle HC Greenwood BM Etiology of acute lower respiratory tract infections in Gambian children: I. Acute lower respiratory tract infections in infants presenting at the hospital Pediatr Infect Dis J 1991 10 1 33 41 10.1097/00006454-199101000-00008 1848364

[B36] Niang M Diop O Sarr F Goudiaby D Malou-Sompy H Ndiaye K Vabret A Baril L Viral etiology of respiratory infections in children under 5 years old living in tropical rural areas of Senegal: The EVIRA project J Med Virol 2010 82 5 866 872 10.1002/jmv.21665 20336732 PMC7166331

[B37] Hall CB Respiratory syncytial virus and parainfluenza virus N Eng J Med 2001 344 25 1917 1928 10.1056/NEJM200106213442507 11419430

[B38] Henrickson KJ Parainfluenza Viruses Clin Microbiol Rev 2003 16 2 242 264 10.1128/CMR.16.2.242-264.2003 12692097 PMC153148

[B39] Cherian T Simoes EAF Steinhoff MC Chitra K John M Raghupathy P John TJ Bronchiolitis in Tropical South India Am J Dis Child 1990 144 9 1026 1030 2396617 10.1001/archpedi.1990.02150330086028

[B40] John TJ Cherian T Steinhoff MC Simoes EAF John M Etiology of Acute Respiratory Infections in Children in Tropical Southern India Review of infectious diseases 1991 13 6 463 469 10.1093/clinids/13.Supplement_6.S463 1862277

[B41] Adegbola R Obaro S Diagnosis of childhood pneumonia in the tropics Ann Trop Med Parasitol 2000 94 3 197 207 10.1080/00034980050006366 10884863

[B42] Kim PE Musher DM Glezen WP Rodriguez-Barradas MC Nahm WK Wright CE Association of invasive pneumococcal disease with season, atmospheric conditions, air pollution, and the isolation of respiratory viruses Clin Infect Dis 1996 22 1 100 106 10.1093/clinids/22.1.100 8824973

[B43] Hishiki H Ishiwada N Fukasawa C Abe K Hoshino T Aizawa J Ishikawa N Kohno Y Incidence of bacterial coinfection with respiratory syncytial virus bronchopulmonary infection in pediatric inpatients J Infect Chemother 2011 17 1 87 90 10.1007/s10156-010-0097-x 20700753

[B44] Berman S Duenas A Bedoya A Constain V Leon S Borrero I Murphy J Acute lower respiratory tract illnesses in Cali, Colombia: a two-year ambulatory study Pediatrics 1983 71 2 210 6823422

[B45] Nwankwo MU Dym AM Schuit KE Offor E Omene JA Seasonal variation in respiratory syncytial virus infections in children in Benin-City, Nigeria Trop Geogr Med 1988 40 4 309 313 3265812

[B46] Kuypers J Wright N Morrow R Evaluation of quantitative and type-specific real-time RT-PCR assays for detection of respiratory syncytial virus in respiratory specimens from children J Clin Virol 2004 31 2 123 129 10.1016/j.jcv.2004.03.018 15364268 PMC7128826

[B47] Heim A Ebnet C Harste G Pring-Akerblom P Rapid and quantitative detection of human adenovirus DNA by real-time PCR J Med Virol 2003 70 2 228 239 10.1002/jmv.10382 12696109

[B48] Kuypers J Wright N Ferrenberg J Huang ML Cent A Corey L Morrow R Comparison of real-time PCR assays with fluorescent-antibody assays for diagnosis of respiratory virus infections in children J Clin Microbiol 2006 44 7 2382 2388 10.1128/JCM.00216-06 16825353 PMC1489473

[B49] van Elden LJ Nijhuis M Schipper P Schuurman R van Loon AM Simultaneous detection of influenza viruses A and B using real-time quantitative PCR J Clin Microbiol 2001 39 1 196 200 10.1128/JCM.39.1.196-200.2001 11136770 PMC87701

